# All three IP_3_ receptor subtypes generate Ca^2+^ puffs, the universal building blocks of IP_3_-evoked Ca^2+^ signals

**DOI:** 10.1242/jcs.220848

**Published:** 2018-08-23

**Authors:** Stefania Mataragka, Colin W. Taylor

**Affiliations:** Department of Pharmacology, University of Cambridge, Tennis Court Road, Cambridge CB2 1PD, UK

**Keywords:** Ca^2+^ puff, Endoplasmic reticulum, IP_3_ receptor subtype, Total internal reflection fluorescence microscopy

## Abstract

All three subtypes of inositol 1,4,5-trisphosphate receptor (IP_3_R) are intracellular Ca^2+^ channels that are co-regulated by IP_3_ and Ca^2+^. This allows IP_3_Rs to evoke regenerative Ca^2+^ signals, the smallest of which are Ca^2+^ puffs that reflect the coordinated opening of a few clustered IP_3_Rs. We use total internal reflection microscopy (TIRF) microscopy to record Ca^2+^ signals in HEK cells expressing all three IP_3_R subtypes or a single native subtype. Ca^2+^ puffs are less frequent in cells expressing one IP_3_R subtype, commensurate with them expressing fewer IP_3_Rs than wild-type cells. However, all three IP_3_R subtypes generate broadly similar Ca^2+^ puffs with similar numbers of IP_3_Rs contributing to each. This suggests that IP_3_R clusters may be assembled by conserved mechanisms that generate similarly sized clusters across different IP_3_R expression levels. The Ca^2+^ puffs evoked by IP_3_R2 had slower kinetics and more prolonged durations, which may be due to IP_3_ binding with greater affinity to IP_3_R2. We conclude that Ca^2+^ puffs are the building blocks for the Ca^2+^ signals evoked by all IP_3_Rs.

## Abstract (precis)

**Summary:** All IP_3_ receptor subtypes can generate Ca^2+^ puffs, suggesting that these coordinated openings of clustered IP_3_Rs are the building blocks of all IP_3_-evoked Ca^2+^ signals.

## INTRODUCTION

Many receptors evoke Ca^2+^ signals by stimulating phospholipase C (Berridge, 2016). Inositol 1,4,5-trisphosphate (IP_3_) then evokes Ca^2+^ release from the endoplasmic reticulum by stimulating IP_3_ receptors (IP_3_Rs), which are widely expressed Ca^2+^ channels. IP_3_ binding to IP_3_R initiates its activation by promoting the Ca^2+^ binding that leads to channel opening (Prole and Taylor, 2016). This dual regulation allows IP_3_Rs to evoke regenerative Ca^2+^ signals, the smallest of which are Ca^2+^ puffs (Smith and Parker, 2009). These arise from the nearly simultaneous opening of a few clustered IP_3_Rs as Ca^2+^ released by one IP_3_R ignites the activity of its neighbours. Ca^2+^ puffs are thought to be fundamental building blocks of IP_3_-evoked Ca^2+^ signalling. They require assembly of IP_3_Rs into small clusters and their anchoring at sites close to the plasma membrane (Smith et al., 2009a) where additional signals prime or ‘license’ IP_3_Rs to respond (Thillaiappan et al., 2017).

Vertebrate genomes encode three IP_3_R subtypes (IP_3_R1, IP_3_R2, IP_3_R3). Each forms a Ca^2+^ channel that is co-regulated by IP_3_ and Ca^2+^, but the subtypes differ in their expression patterns (Taylor et al., 1999), affinities for IP_3_ (Iwai et al., 2007) and modulation by additional signals (Prole and Taylor, 2016). The differences are consistent with evidence that implicates IP_3_R subtypes in specific cellular responses (Miyakawa et al., 1999; Wei et al., 2009), the distinctive phenotypes of mice lacking single subtypes (Futatsugi et al., 2005) and with different diseases arising from mutations in different subtypes (Hisatsune and Mikoshiba, 2017). However, many of these associations might be more related to the predominance of IP_3_R subtypes in different tissues than to fundamental differences in the behaviour of IP_3_R subtypes. Ca^2+^ puffs have been reported for cells in which different IP_3_R subtypes predominate (Keebler and Taylor, 2017), but it is unknown whether all three IP_3_R subtypes can generate Ca^2+^ puffs.

Here, we use total internal reflection fluorescence microscopy (TIRFM) to record Ca^2+^ puffs evoked by photolysis of caged-IP_3_ (ci-IP_3_) in human embryonic kidney 293 (HEK) cells expressing one or all three IP_3_R subtypes. We demonstrate that all three IP_3_R subtypes generate Ca^2+^ puffs with broadly similar properties. We conclude that Ca^2+^ puffs are the fundamental building blocks for Ca^2+^ signals evoked by all IP_3_Rs.

## RESULTS AND DISCUSSION

### Cells with single IP_3_R subtypes generate Ca^2+^ puffs

**Fig. 1.**
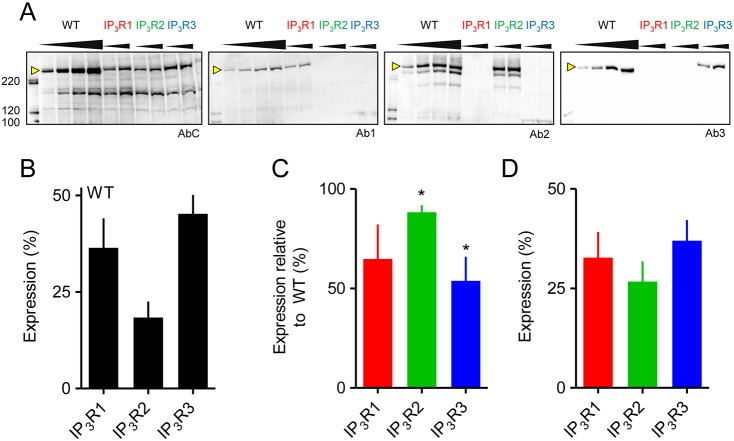
**Expression of IP_3_R subtypes in HEK cells.** (A) Western blots using IP_3_R subtype-selective antibodies (Ab1, Ab2, Ab3) or a common antibody (AbC). Lanes were loaded with 20, 40, 80 or 120 μg protein for WT cells, and with 80 or 120 μg for other cell lines. Molecular mass markers (kDa) are shown. Arrowheads indicate IP_3_R bands used for quantification. (B–D) Summary of results (in %, mean±s.e.m., *n*=5) show relative expression of IP_3_R subtypes in WT cells (B), determined by calibrating bands identified by Ab1, Ab2 or Ab3 to bands identified by AbC in cell lines expressing single IP_3_R subtypes; IP_3_R expression (in %) in cell lines expressing single subtypes relative to the same IP_3_R subtype in WT cells (C); and total number of IP_3_Rs (in %) expressed in each cell line relative to number in WT (D), by comparing intensities of protein bands recognised by AbC. In C, the asterisk (*) denotes values where 95% confidence interval does not include 100%. No significant differences (*P*<0.05) were observed between values in D.

We used wild-type (WT) HEK cells, which express three IP_3_R subtypes (Fig. 1), and HEK cells where CRISPR/Cas9 had been used to generate cells expressing single subtypes (Alzayady et al., 2016). Identical stimuli were delivered to each cell by photolysis of ci-IP_3_, which uniformly releases the active, but slowly degraded, analogue of IP_3_, iIP_3_ (d-2,3-*O*-isopropylidine-IP_3_) (Dakin and Li, 2007). We confirmed, by recording Ca^2+^ signals evoked by photolysis of caged-Ca^2+^, that Ca^2+^ buffering was similar in each cell line (Fig. S1A,B).

Antibodies selective for each IP_3_R subtype (Ab1–3) confirmed that WT HEK cells express three IP_3_R subtypes (IP_3_R3≥IP_3_R1>IP_3_R2; Fig. 1A,B). This profile differs from previous analyses using immunoblots (Wojcikiewicz, 1995) or QPCR (Tovey et al., 2008), which suggested IP_3_R1 to be the minor subtype. The differences might be due to the methods used or the source of HEK cells – our cell line is the one from which the gene-edited cells were derived. Within each cell line expressing a single IP_3_R subtype, its expression level was lower than in WT cells (Fig. 1A,C). By using an antibody that recognises a sequence conserved in all IP_3_R subtypes (AbC), we established that overall IP_3_R expression levels were similar in each cell line expressing a single IP_3_R subtype (Fig. 1D), but lower (∼32%) than in WT cells (Fig. 1D).

**Fig. 2.**
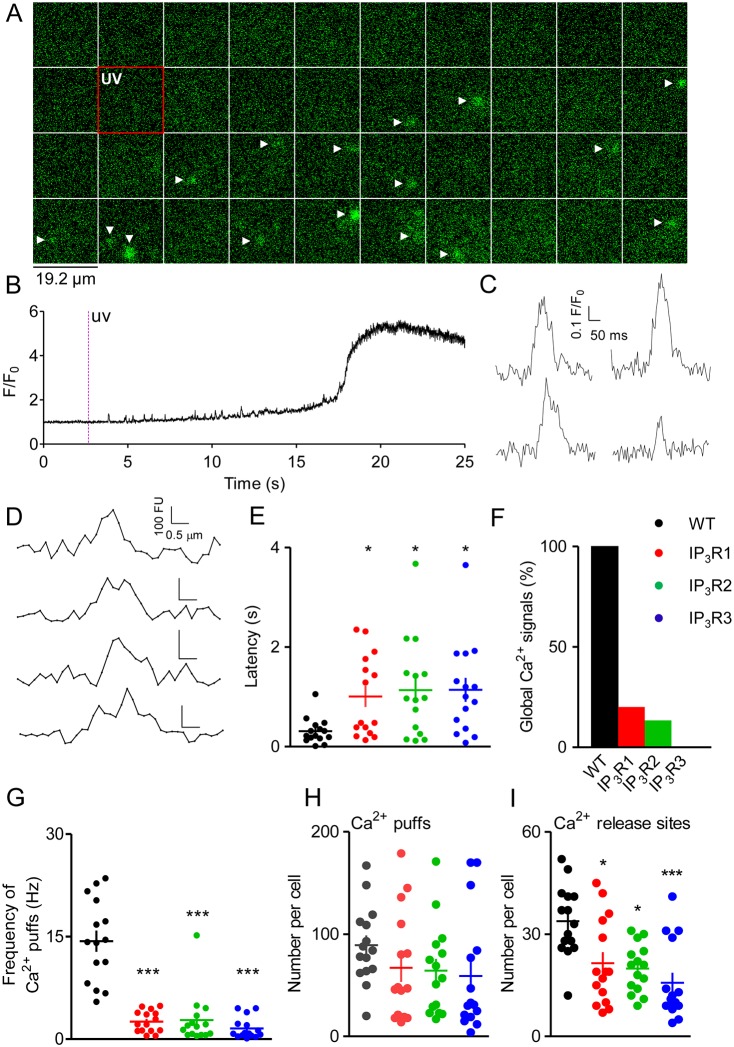
**Ca^2+^ puffs evoked by photolysis of ci-IP_3_.** TIRFM images from part of a WT HEK cell show fluorescence before and after photolysis of ci-IP_3_. The first frame after the UV flash has a red border. Images were collected at 5.32-ms intervals, with every 20th frame shown. Arrowheads show Ca^2+^ puffs identified by Flika image processing. (B) Fluorescence (F/F_0_) recorded before and after the UV flash shows Ca^2+^ puffs before the Ca^2+^ signal propagates globally (recording area 1.76×1.76 μm). (C,D) Time courses of Ca^2+^ puffs in WT cells (C) and fluorescence profiles at the peak of each puff (D). (E) Latencies to first detected Ca^2+^ puff. (F) Cells (in %) that showed a global increase in [Ca^2+^]_c_ within the 40-s recording. (G–I) Frequency of Ca^2+^ puffs (G), total numbers of Ca^2+^ puffs (H) and release sites (I) detected during 40-s recording. In E and G-I, results show individual mean values from 15 cells, and the mean±s.e.m. of those values. *P<0.05, ***P<0.001, relative to WT. The colour code used in E–I is explained in F.

In WT cells, but not in HEK cells without IP_3_Rs (Fig. S1C), photolysis of ci-IP_3_ evoked Ca^2+^ puffs after a short latency. The Ca^2+^ signal then propagated after a few seconds to produce a global increase in cytosolic free Ca^2+^ concentration ([Ca^2+^]_c_) (Fig. 2A–D). These responses are similar to those observed in other cell types (see Keebler and Taylor, 2017 and references therein). However, and consistent with our analyses of HEK and HeLa cells (Keebler and Taylor, 2017; Thillaiappan et al., 2017), the sites at which Ca^2+^ puffs occurred in the 40 s after photo-release of iIP_3_ (33.8±2.7 sites, *n*=15 cells; Fig. 2I) were about ten-times more abundant than reported in studies using different cell types (Nakamura et al., 2012; Smith and Parker, 2009; Thomas et al., 2000). Our detection of more Ca^2+^ release sites is not due to mis-identification of Ca^2+^ puffs but probably arises from improved detection through automated analysis and longer effective recordings (Keebler and Taylor, 2017).

Photolysis of ci-IP_3_ also evoked Ca^2+^ puffs in HEK cells expressing single IP_3_R subtypes. In these cell lines, all of which were exposed to the same stimulus, the latency before the first Ca^2+^ puff was longer than in WT cells, Ca^2+^ puffs were less frequent, we detected fewer Ca^2+^-release sites and it was rare for Ca^2+^ signals to propagate into a global increase in [Ca^2+^]_c_ (Fig. 2E–I).

### Ca^2+^ puffs initiate at fewer sites in cells expressing only one IP_3_R subtype

In HEK WT cells (Keebler and Taylor, 2017), as in other cell types (Smith et al., 2009b; Thillaiappan et al., 2017), Ca^2+^ release sites remain at fixed locations for many minutes. The opportunity to identify Ca^2+^ release sites depends on the number of Ca^2+^ puffs: more puffs provide more opportunities to find sites. Ca^2+^ puffs were frequent in WT cells, but were detected for only a few seconds before the Ca^2+^ signal invaded the cell (Fig. 2B,F). In the other HEK cell lines used by us, Ca^2+^ puffs were less frequent (Fig. 2G) but the useful recording interval was longer because Ca^2+^ signals rarely became global (Fig. 2F, Fig. S1D). Fortuitously, the average number of Ca^2+^ puffs detected during the entire recording interval was not significantly different between the four cell lines, but we detected significantly more Ca^2+^ release sites in WT HEK cells (Fig. 2H,I).

Another approach to identifying the total number of release sites is to recognise that, as the recording proceeds, each successive Ca^2+^ puff has a diminished opportunity to reveal a new site. For cells expressing single IP_3_R subtypes, we divided the 40-s recording into 10-s intervals, in each of which a similar number of Ca^2+^ puffs was detected (Fig. S2A). The number of new sites identified in each successive interval declined mono-exponentially with half-times (t_1/2_=3.76–5.53 s) (Fig. S3A) that were fast enough to ensure that, within the 40-s recording interval (>4 half-lives), we should identify >94% of all Ca^2+^ release sites. The analysis of WT cells used a shorter time-bin (2 s) to accommodate the increased frequency of Ca^2+^ puffs (Fig. 2G) but yielded similar results. The typical effective recording period (∼10 s) for WT cells was about four-times longer than the half-time for the mono-exponential decay of the discovery of new Ca^2+^ release sites (t_1/2_=2.01 s), indicating that we should again detect >93% of sites (Fig. S3B). Hence, for all cell lines our analyses are likely to detect most Ca^2+^ release sites. The fewer sites identified in cells with single IP_3_R subtypes are, therefore, not due to inadequate sampling. We conclude that the cell lines with single IP_3_R subtypes express about one-third the total number of IP_3_Rs of WT cells (Fig. 1D) and about half the number of Ca^2+^-release sites (Fig. 2I).

### Ca^2+^ puffs evoked by IP_3_R2 are more long-lasting

There is considerable variation in the shape and duration of Ca^2+^ puffs (Fig. 2C,D, Fig. S1E) (Smith and Parker, 2009). For Ca^2+^ puffs in WT HEK cells, the fluorescence signals typically rose rapidly to a peak before more slowly decaying back to baseline (Fig. 2C). To secure sufficient data for quantitative analysis, we analysed Ca^2+^ puffs for as long as practicable within the 40-s recording. For WT cells this entailed recording frequent Ca^2+^ puffs for the relatively short interval (typically ∼10 s) before the Ca^2+^ signal invaded the cell, whereas – for the other cell lines – it entailed recording less frequent Ca^2+^ puffs for longer periods (40 s) (Fig. 2F,G). To validate comparisons of WT cells with the other cell lines, we showed there were no systematic changes in Ca^2+^ puff frequency across the analysis intervals (Fig. S2A).

**Fig. 3.**
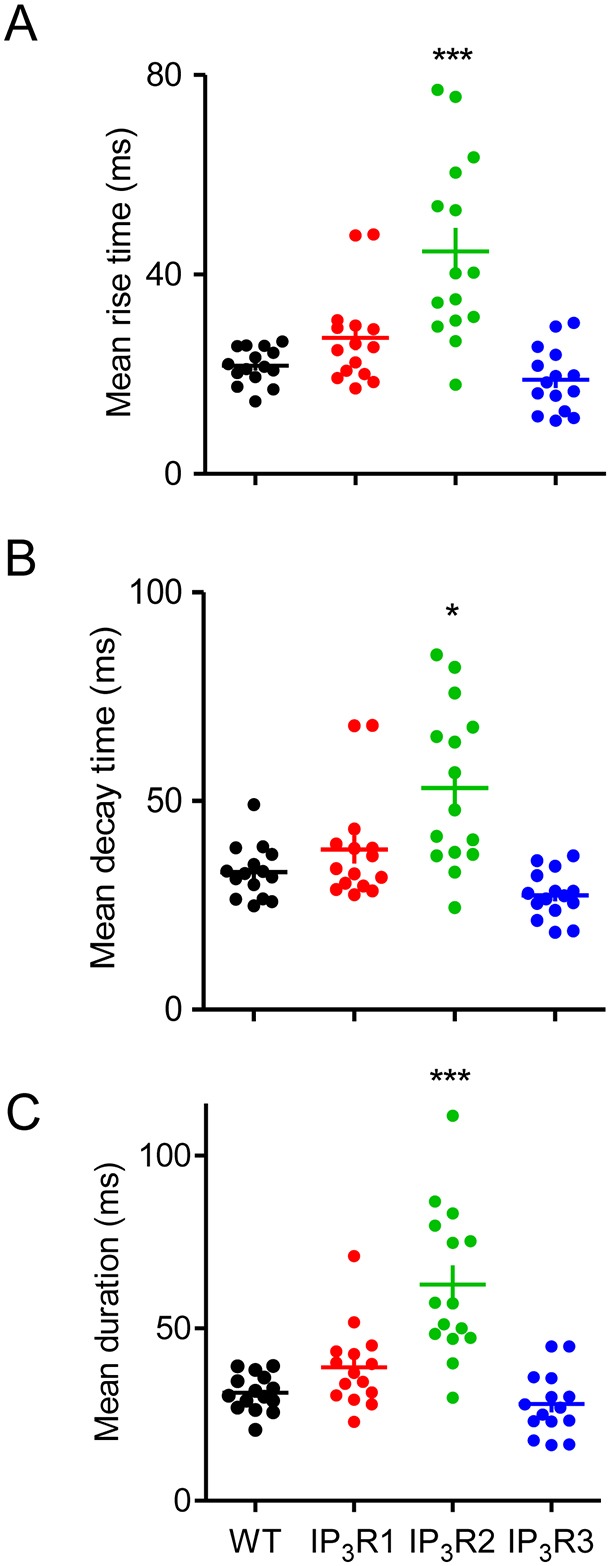
**Properties of Ca^2+^ puffs in cells expressing a single IP_3_R subtype.** (A–C) Mean rise time (A) – i.e. time for fluorescence to increase from 20% to 100% of peak, decay time (B) – i.e. time to fall from 100% to 20%, and mean duration at half-maximal amplitude (C) of Ca^2+^ puffs evoked by photolysis of ci-IP_3_. Results show mean values for each of 15 cells for each cell line (∼1000 puffs per cell line were analysed), and mean±s.e.m. of these values. *P<0.05, ***P<0.001, relative to WT.

We compared mean rise times, decay times and durations of the Ca^2+^ puffs in the four cell lines (Fig. 3A–C) and their frequency distributions (Fig. S4). The rise times (∼20 ms), decay times (∼30 ms) and durations at half-maximal amplitude (∼30 ms) were similar in WT cells and in cells expressing only IP_3_R1 or IP_3_R3. However, the rise and decay times (44.6±4.7 ms and 53.1±5.0 ms, respectively) were significantly longer in cells expressing only IP_3_R2, as was the mean duration of Ca^2+^ puffs (62.6±5.6 ms) (Fig. 3). The frequency distributions revealed that the more-prolonged events in cells expressing IP_3_R2 were largely attributable to an extended tail in the distribution: very long events rarely seen in other cell lines were more frequent in IP_3_R2-expressing cells (Fig. S4). We conclude that all three IP_3_R subtypes generate Ca^2+^ puffs with broadly similar characteristics, although Ca^2+^ puffs evoked by IP_3_R2 typically last about twice as long as those in cells expressing IP_3_R1, IP_3_R3 or all three subtypes.

### A similar number of IP_3_Rs contributes to Ca^2+^ puffs for each IP_3_R subtype

**Fig. 4.**
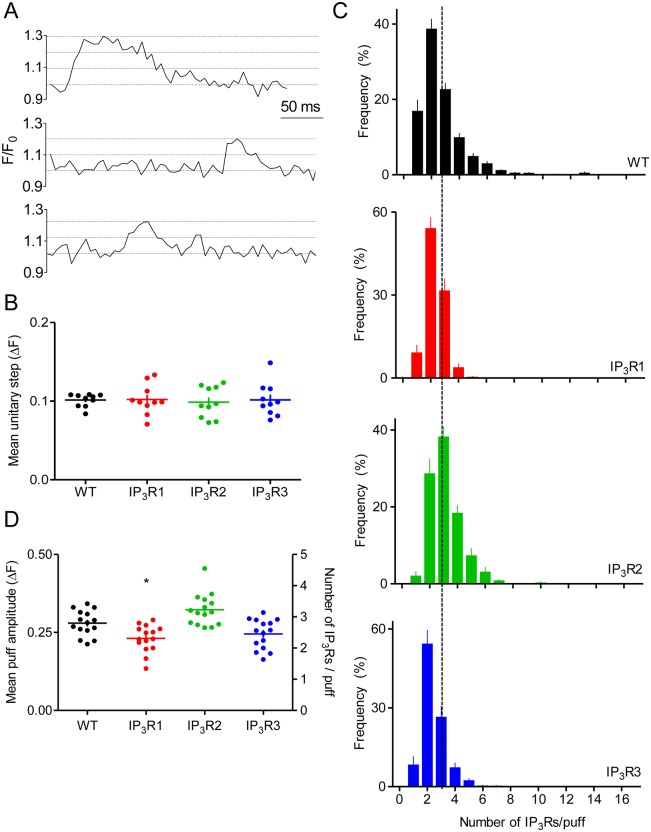
**Amplitudes of Ca^2+^ puffs evoked by different IP_3_R subtypes.** (A) Examples of Ca^2+^ puffs from WT cells show discrete fluorescence steps in falling phase. Dashed lines show unitary steps. (B) Summary of results (mean±s.e.m. *n*=10 puffs from different cells) show the amplitude of unitary fluorescence steps during the falling phase of Ca^2+^ puffs. Values do not differ significantly between cell lines (*P*>0.05). (C) Frequency distributions (mean±s.e.m., *n*=15 cells) show the estimated number of active IP_3_Rs within each Ca^2+^ puff. The dashed line indicates the mean value for WT cells. (D) Summary of results (mean±s.e.m., *n*=15 cells) show the ΔF values from which the number of active IP_3_Rs within each Ca^2+^ puff was calculated. *P<0.05, relative to WT.

During the falling phase of a Ca^2+^ puff, it was sometimes possible to observe stepwise closures of individual IP_3_Rs (Fig. 4A, Fig. S1E) (Smith and Parker, 2009). The amplitude of these unitary fluorescence steps was indistinguishable in the four cell lines (Fig. 4B), suggesting that each IP_3_R subtype is similarly distributed within the TIRF field. The results are also consistent with evidence that IP_3_R subtypes have a similar cation conductance (Vais et al., 2010). We used the average mean amplitude of the unitary fluorescence step from all cells (ΔF=0.101±0.002, *n*=40) and the peak amplitude of each Ca^2+^ puff, to estimate the number of IP_3_Rs contributing to a Ca^2+^ puff (Smith and Parker, 2009). The results show that most Ca^2+^ puffs involve fewer than seven IP_3_Rs (Fig. 4C, Fig. S5D). In WT cells, the mean number of IP_3_Rs per puff was 2.77±0.10 (Fig. 4C,D). Our results are similar to those from SH-SY5Y cells, for which 50% of IP_3_Rs within a cluster are suggested to open during a typical Ca^2+^ puff (Smith et al., 2009a). Collectively, these observations are consistent with analyses of endogenous IP_3_Rs in HeLa cells, suggesting that IP_3_R clusters include about eight IP_3_Rs (Thillaiappan et al., 2017). The mean peak amplitudes of Ca^2+^ puffs were indistinguishable in WT cells and cells expressing only IP_3_R3. The amplitudes in cells expressing IP_3_R1 and IP_3_R2 were different from those in WT cells (Fig. 4C,D), but the differences were modest (<18%), with an estimated 2.28±0.11 and 3.19±0.13 IP_3_Rs per puff for cells with IP_3_R1 and IP_3_R2, respectively (Fig. 4D). We conclude that, despite differences in IP_3_R expression between WT cells and cells expressing a single IP_3_R subtype (Fig. 1D), Ca^2+^ puffs are generated by a similar number of IP_3_Rs in each cell line (Fig. 4).

We considered whether Ca^2+^ puffs occurring in the most-active cells differed in their properties. There were no clear differences in the frequency distribution for rise time, decay time or Ca^2+^ puff duration, or in the estimated number of IP_3_Rs per puff for the more-active and less-active cells (Fig. S5). We also compared the mean peak amplitude of Ca^2+^ puffs evoked in each cell with mean rise and fall times. Again, there was no significant correlation between puff amplitude and kinetics; the only exception was a positive correlation between amplitude and decay time for cells expressing IP_3_R2 (Fig. S6).

### Conclusions

We show, for the first time, that all three IP_3_R subtypes generate Ca^2+^ puffs with broadly similar properties. Ca^2+^ puffs have hitherto been observed only in cells that express all three IP_3_R subtypes, albeit with one subtype usually predominating. Ca^2+^ puffs originate at fixed intracellular sites, at each of which a loose cluster of a few IP_3_Rs is anchored near the plasma membrane, where an additional signal licenses the cluster to respond to IP_3_ (Smith et al., 2009a; Thillaiappan et al., 2017). Neither the scaffold that assembles clusters nor the signal that licenses them to respond has been identified. WT HEK cells expressed three-times more IP_3_Rs than HEK cells with single IP_3_R subtypes (Fig. 1D), yet each cell line generated Ca^2+^ puffs in which the unitary steps during the falling phase and number of active IP_3_Rs contributing to each Ca^2+^ puff were similar (Fig. 4B-D, Figs S1E, S5D). These results suggest that, first, for each subtype, IP_3_Rs are similarly distributed across the ∼90-nm depth of the TIRF field, consistent with each coming close to the plasma membrane. Second, across a range of IP_3_R expression levels, IP_3_Rs are assembled into similarly sized clusters, but with more clusters in cells with more IP_3_Rs (Fig. 2I, Fig. S3). We conclude that the clusters of IP_3_Rs that generate Ca^2+^ puffs are probably not random associations. Instead, IP_3_Rs appear to be assembled through mechanisms that are conserved for all IP_3_R subtypes, and which ensure that similarly sized clusters are assembled irrespective of IP_3_R expression levels.

The similar properties of Ca^2+^ puffs evoked by cells expressing all IP_3_R subtypes or by those expressing only a single subtype are striking. However, Ca^2+^ puffs evoked by IP_3_R2 were more long-lasting than those evoked by other subtypes. We have not determined the mechanisms responsible for the slower kinetics of Ca^2+^ puffs evoked by IP_3_R2. One possibility is that the greater affinity of IP_3_ for IP_3_R2 (Iwai et al., 2007) slows IP_3_ dissociation and delays channel closures. In *Xenopus* oocytes, however, Ca^2+^ puffs evoked by the high-affinity IP_3_ analogue adenophostin A were briefer than those evoked by IP_3_ (Marchant and Parker, 1998). Furthermore, in our analyses, the frequency of Ca^2+^ puffs evoked by identical stimuli in cell lines expressing IP_3_R1, IP_3_R2 or IP_3_R3 were indistinguishable (Fig. 2G), which seems difficult to reconcile with the idea that IP_3_Rs have substantially different affinities *in situ*. It might be that the mechanism that allows IP_3_R clusters to respond overrides any underlying differences in the IP_3_ affinity of IP_3_R subtypes.

We provide the first demonstration that each IP_3_R subtype generates Ca^2+^ puffs with broadly similar properties, leading us to suggest that the small, loose IP_3_R clusters from which Ca^2+^ puffs originate are the building blocks for the Ca^2+^ signals evoked by all IP_3_Rs.

## MATERIALS AND METHODS

### Materials

Cal520-AM was from Stratech Scientific (Suffolk, UK). Bovine serum albumin (BSA) was from Europa Bio-Products (Ely, UK). Gibco TrypLE Express, NP-EGTA-AM (caged EGTA, *O*-nitrophenyl-EGTA-AM) and Dulbecco's modified Eagle's medium (DMEM)/Ham's F-12 (50:50) supplemented with GlutaMAX were from ThermoFisher (Waltham, MA). Pluronic acid F-127, dimethyl sulfoxide (DMSO), poly-l-lysine and foetal bovine serum were from Sigma-Aldrich (Poole, Dorset, UK). EGTA-AM was from Merck Millipore (Darmstadt, Germany). Caged-IP_3_-PM [ci-IP_3_-PM, d-2,3-*O*-isopropylidine-6-*O*-(2-nitro-4,5-dimethoxy)benzyl-*myo*-inositol 1,4,5-trisphosphate-hexakis (proprionoxymethyl)ester] was from SiChem GmbH (Bremen, Germany). Custom-made rabbit anti-peptide antibodies against IP_3_R2 (Ab2, GFLGSNTPHENHHMPPH, 1:1000) and to a sequence conserved in all three IP_3_R subtypes (AbC, PMRYSAKQKFWKA, 1:500) were provided by Poccono Rabbit Farm and Laboratory Inc. (Canadensis, PA). Additional primary antibodies were: IP_3_R1 (Ab1, rabbit, #3763, Cell Signaling Technology, Leiden, The Netherlands, 1:1000), IP_3_R3 (Ab3, mouse, #610312, BD Biosciences, Wokingham, UK, 1:1000) and β-actin (mouse, #3700, Cell Signaling Technology, 1:1000). Secondary antibodies conjugated to horseradish peroxidase (anti-mouse, sc-516102; anti-rabbit, sc-2357, 1:5000) were from Insight Biotechnology Ltd (Wembley, UK).

### Cell culture

WT HEK cells, from which the gene-edited lines were generated, were provided by Dr D. Yule (University of Rochester, NY). HEK cells in which CRISPR/Cas9 had been used to generate lines expressing a single IP_3_R subtype or devoid of all IP_3_Rs (HEK-KO) (Alzayady et al., 2016) were from Kerafast (Boston, MA). We confirmed that all cells were mycoplasma-free. Cells were maintained in DMEM/Ham's F-12 supplemented with GlutaMAX and 10% foetal bovine serum in humidified air at 37°C with 5% CO_2_, and passaged when they reached ∼80% confluence. For imaging, cells (5×10^4^/dish) were grown on poly-l-lysine-coated (10 µg/ml) glass-bottomed dishes (P35GC-1.5-14-C, MatTek, Ashland, MA). They were used after 48 h, when they were sub-confluent.

### Immunoblotting

Cells were lysed in cold RIPA buffer (1 mM Tris HCl, 15 mM NaCl, 0.5 mM EDTA, 0.1% Triton X, pH 7.5) with protease inhibitors (cOmplete, EDTA-free protease inhibitor cocktail, Sigma-Aldrich). Proteins in the supernatant (900× ***g***, 4 min, 4°C) were separated (NuPAGE 3-8% Tris-acetate protein gel, 1.0 mm, 12-well, ThermoFisher) and transferred to a polyvinyl difluoride (PVDF) membrane using an iBLOT gel-transfer system (ThermoFisher). Membranes were blocked in Tris-buffered saline (TBS: 50 mM Tris-HCl, 150 mM NaCl, pH 7.5, 0.2% Tween-20, 5% BSA (1 h, 20°C) and incubated (40 h, 4°C) with primary antibody. After three 5-min washes in TBS, membranes were incubated (1 h, 20°C) with secondary antibody and washed (3×5 min). Bands were visualised with ECL Primer Western blotting detection reagent (SLS, Nottingham, UK) and a Syngene PXi chemiluminescence detection system (Cambridge, UK). We confirmed that, within the range of loadings used for analysis, IP_3_R band intensities scaled linearly with the amount of protein loaded.

To quantify expression of IP_3_R subtypes in WT HEK cells, we used the cells expressing single subtypes to determine the ratio of band intensities for each IP_3_R subtype-selective antibody (Ab1-3) relative to the common antibody (e.g. Ab1:AbC for IP_3_R1). These ratios were used to convert intensities of IP_3_R subtype-selective bands in WT cells to AbC intensities, from which we estimated the relative expression of the three IP_3_R subtypes (Fig. 1B).

### High-resolution imaging of Ca^2+^ signals

To record local Ca^2+^ signals, cells were loaded with EGTA to restrict Ca^2+^ diffusion (Parker and Smith, 2010), a Ca^2+^ indicator (Cal520) and ci-IP_3_. Cells were washed with HEPES-buffered saline (HBS; 135 mM NaCl, 5.9 mM KCl, 1.2 mM MgCl_2_, 1.5 mM CaCl_2_, 11.5 mM glucose, 11.6 mM HEPES pH 7.3) and then incubated (20°C in darkness) in HBS containing pluronic acid F-127 (0.02% w/v), Cal520-AM (5 µM) and ci-IP_3_-PM (1 µM). After 1 h, cells were washed and incubated in HBS containing pluronic acid F-127 and EGTA-AM (5 µM). After 45 min, the medium was replaced with HBS. After a further 30 min, the cells in HBS at 20°C, were used for imaging. The same method, but with NP-EGTA-AM (1 μM) replacing ci-IP_3_ PM, was used to load cells with caged EGTA (Fig. S1A,B).

An Olympus IX83 inverted microscope equipped with 100× oil-immersion TIRF objective (Olympus UApo N; numerical aperture, NA=1.49) was used for measurements of local Ca^2+^ signals by using total internal reflection fluorescence microscopy (TIRFM). Excitation (488 nm) was provided by a diode-pumped solid-state laser (150 mW, iLas Laser System, Cairn) and a band-pass filter (ET-405/488/561/643 quad band filter set, Chroma). The angle of the excitation beam was adjusted to achieve TIRF with a penetration depth of ∼90 nm. Emitted light was captured through a band-pass filter (Cairn Optospin, peak/bandwidth 525/50 nm) using an Andor iXon 897 EMCCD camera (image size: 120×120 pixels, 16 µm×16 µm per pixel; each image pixel had dimensions of 160 nm×160 nm). To achieve high temporal resolution, images were captured (188 Hz) from only a part of each cell (19.2 µm×19.2 µm, ∼25% of its TIRF footprint; see Fig. S2B,C) by streaming directly into RAM. After visualisation using Metamorph (Molecular Devices, Sunnyvale, CA), images were exported as tif files. Images were captured for 40 s before and after flash photolysis of ci-IP_3_.

Photolysis of ci-IP_3_ or NP-EGTA within the entire imaging field was achieved using a light-emitting diode (LED, 395 nm, Spectra Lumencor, Beaverton, OR), with the duration of exposure (50 ms, for ci-IP_3_; 300 or 1000 ms for NP-EGTA) determined by a shutter controlled within Metamorph. The conditions used for flash photolysis of ci-IP_3_ were optimised to allow identical stimulation of all cell lines, with each line providing sufficient Ca^2+^ puffs for quantitative analysis within a practicable recording interval (≤40 s).

### Detection and analysis of Ca^2+^ puffs

We confirmed that the recording areas were similar for the four cell lines examined (Fig. S2C). Images were processed in Fiji (Schindelin et al., 2012). Images were corrected for background by subtraction of the fluorescence recorded from a region of interest (ROI) outside the cell. For automated analysis of Ca^2+^ puffs, the records for 2.7 s before and 40 s after photolysis were loaded into the image-processing program flika (Ellefsen et al., 2014) (https://github.com/flika-org/flika) that identifies clusters of pixels within which fluorescence changes exceed a critical threshold. From each recording, the last 500 frames (∼2.7 s) before photolysis were averaged to provide an F_0_ value and its standard deviation (s.d.) for each pixel. Image stacks of [(F/F_0_ )−1]/s.d. were then created and Gaussian-filtered, and pixels that exceeded a critical value (0.8 for our analysis) were located. A threshold-cluster algorithm (Rodriguez and Laio, 2014) then identified cluster centres by comparing them with adjacent bright pixels. Flika then identified the frames within which the event occurred and 100 frames either side of it. Fluorescence values of each pixel within a ROI (1.76×1.76 μm) centred on the brightest pixel were then averaged for each frame and a 2D Gaussian function was fitted to locate the centre of each Ca^2+^ puff. We confirmed, by manual inspection, the reliability and effectiveness of the automated identification of Ca^2+^ puffs. Flika then catalogued properties of the Ca^2+^ puffs, and assigned each Ca^2+^ puff to a Ca^2+^ release site. In our analysis, we considered Ca^2+^ puffs to arise at different sites when the centroids of the Ca^2+^ puffs were more than 0.96 μm apart.

To estimate the number of active IP_3_Rs contributing to a Ca^2+^ puff (*N*), we measured the unitary steps during the decay phase of 40 Ca^2+^ puffs (ten from each cell line, Fig. 4B). The average value of this unitary step (0.101±0.002) was used to estimate *N* from: *N* = (ΔF/0.101), where ΔF = F_peak_−F_pre_. F_peak_ and F_pre_ are, respectively, the F/F_0_ values determined at the peak of the Ca^2+^ puff and the average from the same region for ten frames before and after the puff.

### Statistical analyses

Results are presented as means±s.e.m. unless stated otherwise, with *n* usually referring to the number of cells analysed. Statistical analyses used two-tailed Mann–Whitney tests or, for multiple comparison, the Kruskal–Wallis test with Dunn's correction for multiple comparisons (PRISM version 6, GraphPad, CA). Statistical significance is denoted by: *P<0.05, **P<0.01, ***P<0.001.

## Supplementary Material

Supplementary information
