# Supplementary material for: All three IP3 receptor subtypes generate Ca2+ puffs, the universal building blocks of IP3-evoked Ca2+ signals
Source: J Cell Sci. 2018 Aug 23;131(16):jcs220848. doi: 10.1242/jcs.220848 (PMC6127726; doi:10.1242/jcs.220848)
Supplement: Supplementary information [file joces-131-220848-s1.pdf]

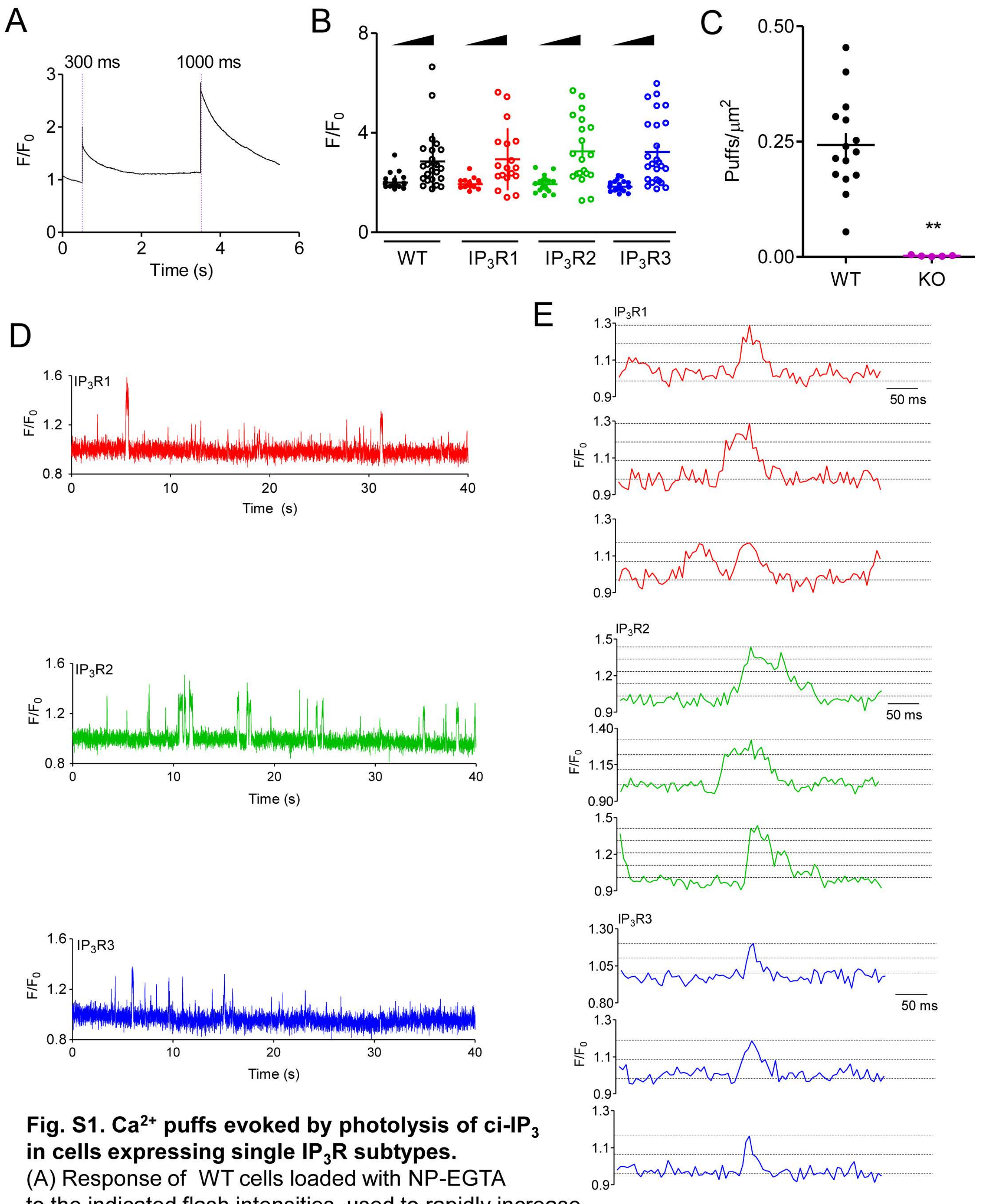

Fig. S1. Ca^{2+} puffs evoked by photolysis of ci-IP_3 in cells expressing single IP_3R subtypes.
(A) Response of WT cells loaded with NP-EGTA to the indicated flash intensities, used to rapidly increase $[Ca^{2+}]_c$ (recorded from a ROI of 1.76 x 1.76 μm, n = 24 cells). (B) Summary results (mean ± s.d. and individual values, n = 18-25 cells) show peak fluorescence changes evoked by flash durations of 300 ms (filled circles) or 1000 ms (open circles). (C) Numbers of Ca^{2+} puffs detected in the 40 s after photolysis of ci-IP_3 in WT cells and HEK cells without IP_3Rs (KO). Results show individual values from 5 (KO) and 15 (WT) cells, and mean ± s.e.m. $^{**}P < 0.01$, two-tailed Mann-Whitney test. (D) Typical fluorescence changes (F/F_0) recorded from areas (1.76 x 1.76 μm) within cells expressing each of the indicated IP_3R subtypes. (E) Examples of Ca^{2+} puffs from each cell line, with unitary steps in the falling phase shown by dashed lines.

Journal of Cell Science • Supplementary information

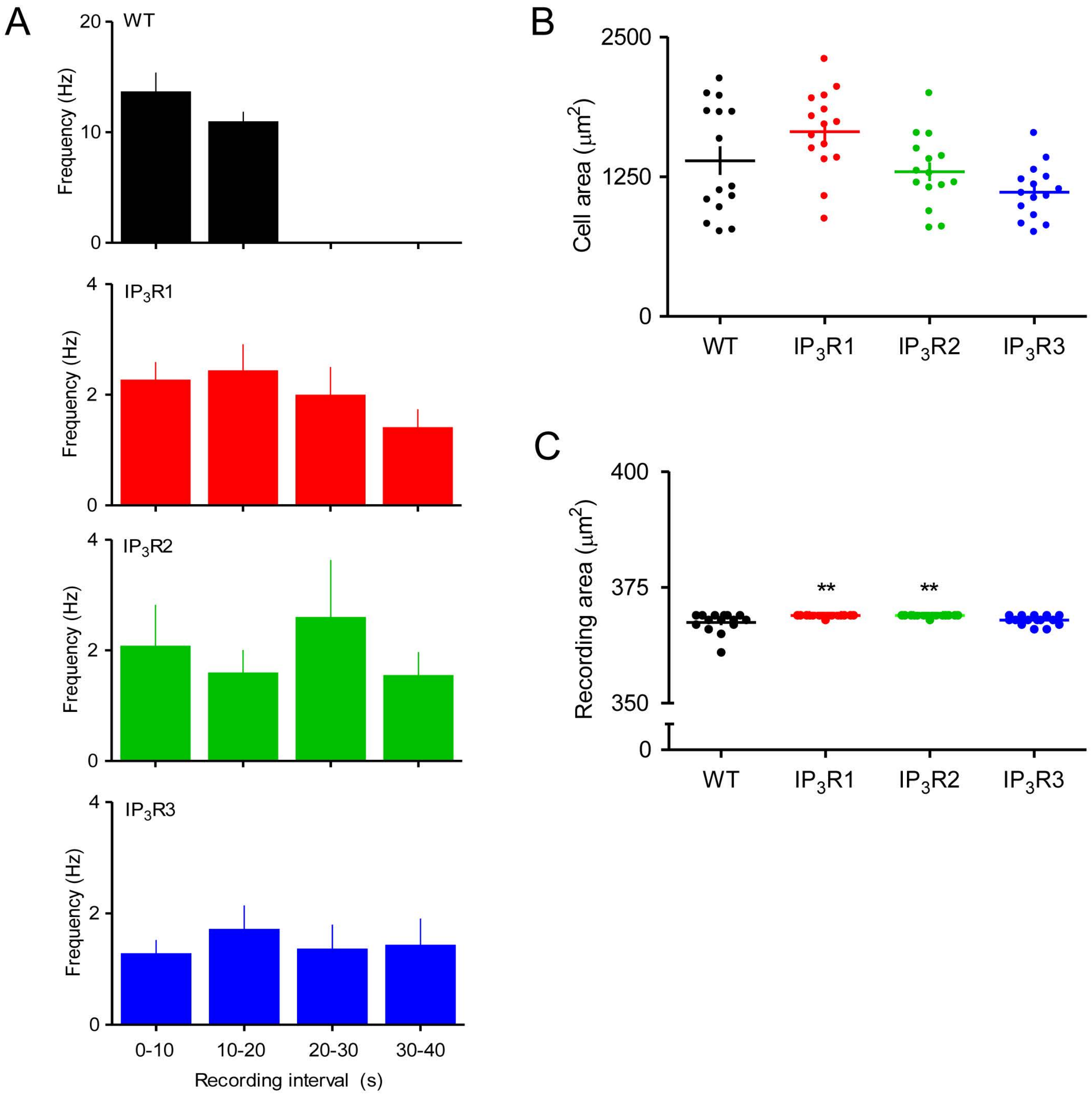

Fig. S2. Ca^{2+} puffs occur with uniform frequency throughout the recording period and are recorded from similar areas for all four cell lines. (A) Our comparison of Ca^{2+} puffs across cell lines necessitated analysis of events taken from systematically different post-stimulation intervals for WT cells (typically ~10 s) and the other cell lines (usually 40 s). Although i-IP_3, the IP_3 analogue released after photolysis of ci-IP_3, is more metabolically stable than IP_3 (Dakin and Li, 2007), we were concerned that it might have been degraded more in the more prolonged analyses of the cell lines expressing single IP_3R subtypes relative to the shorter analyses of WT cells. Ca^{2+} signals were evoked by photolysis of ci-IP_3 in each of the cell lines, and the frequency of Ca^{2+} puffs (Hz) was measured for each interval beginning after the average latency for each cell line (**Fig. 2E**) until the recording was terminated at either 40 s or when the Ca^{2+} signal invaded the cell. There were no significant differences between values determined in the later time intervals relative to the first interval ($P > 0.05$). (B,C) Total cell areas detected by TIRFM (B) and recording areas within the TIRF field (C) for each cell line. Results show individual values ($n = 15$ for each cell line) and the mean ± s.d. ** $P < 0.01$ relative to WT. Although the recording areas for cells expressing IP_3R1 or IP_3R2 were significantly different from WT cells, the differences were <0.4% of the average recording area and not therefore a source of experimental error. The results validate our comparisons of the four cell lines.

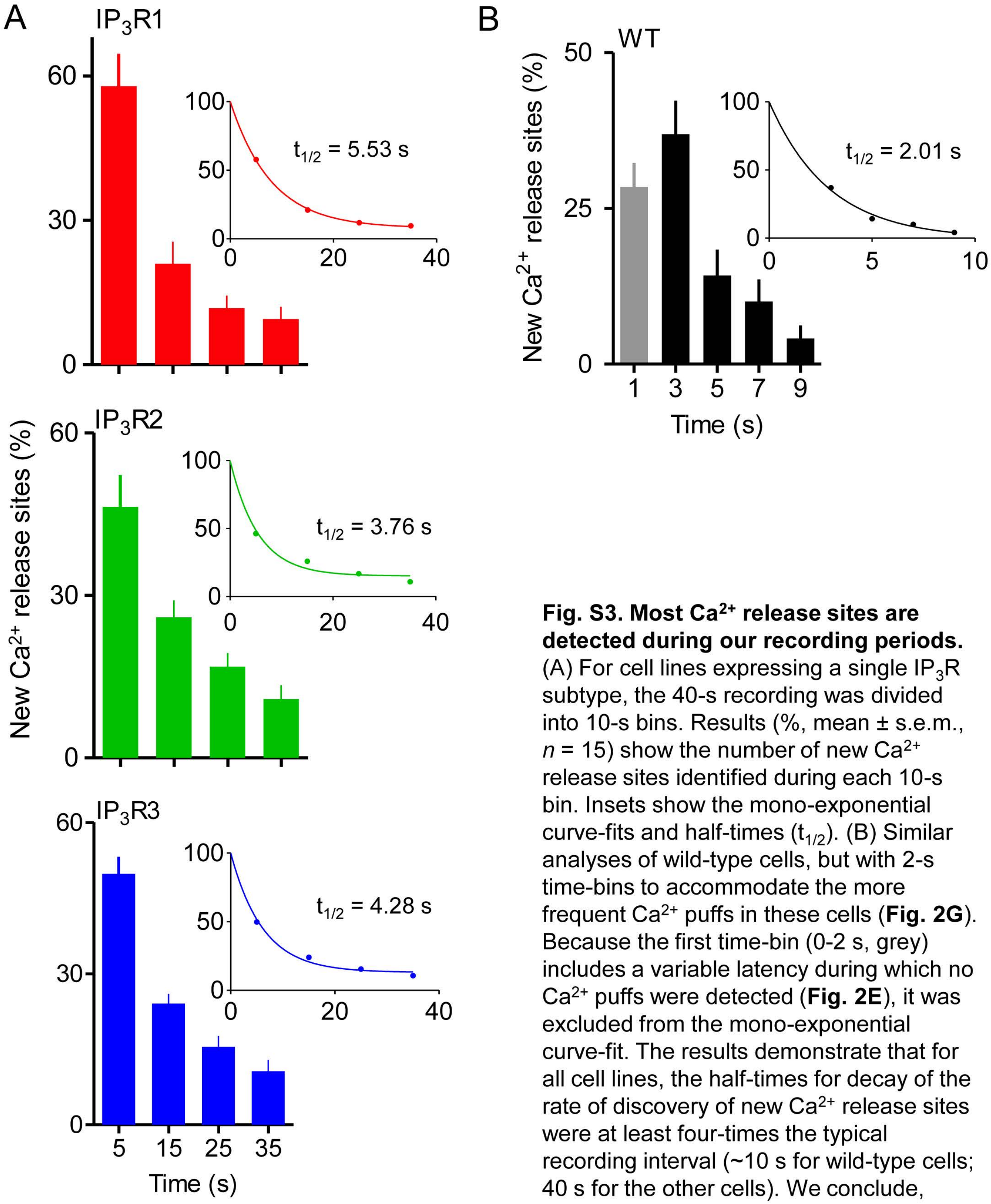

Fig. S3. Most Ca^{2+} release sites are detected during our recording periods. (A) For cell lines expressing a single IP_3R subtype, the 40-s recording was divided into 10-s bins. Results (%, mean ± s.e.m., *n* = 15) show the number of new Ca^{2+} release sites identified during each 10-s bin. Insets show the mono-exponential curve-fits and half-times ($t_{1/2}$). (B) Similar analyses of wild-type cells, but with 2-s time-bins to accommodate the more frequent Ca^{2+} puffs in these cells (**Fig. 2G**). Because the first time-bin (0-2 s, grey) includes a variable latency during which no Ca^{2+} puffs were detected (**Fig. 2E**), it was excluded from the mono-exponential curve-fit. The results demonstrate that for all cell lines, the half-times for decay of the rate of discovery of new Ca^{2+} release sites were at least four-times the typical recording interval (~10 s for wild-type cells; 40 s for the other cells). We conclude, therefore, that the durations of our recordings (t) were sufficient ($t > 4 \times t_{1/2}$) to detect >90% of all Ca^{2+} release sites.

Journal of Cell Science • Supplementary information

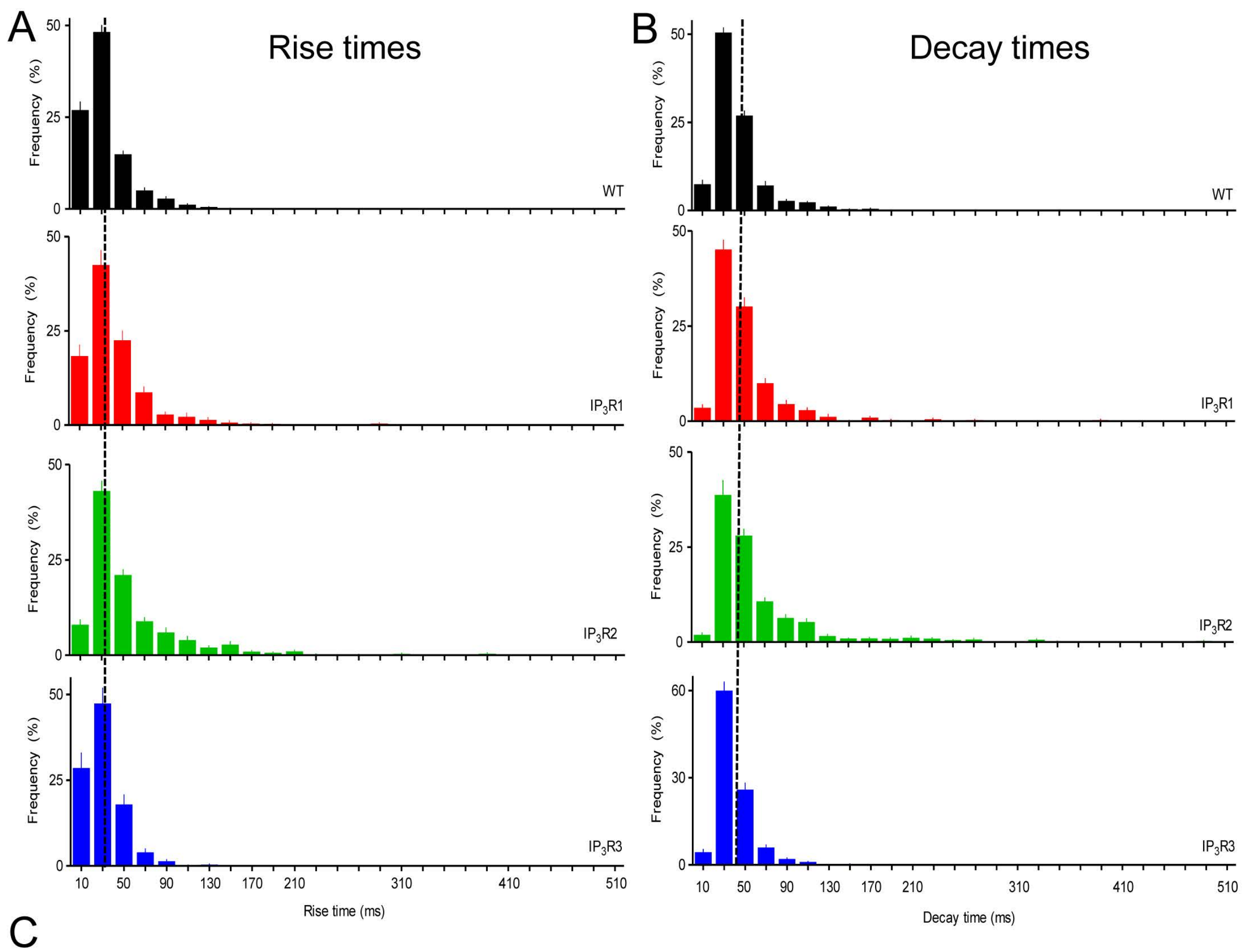

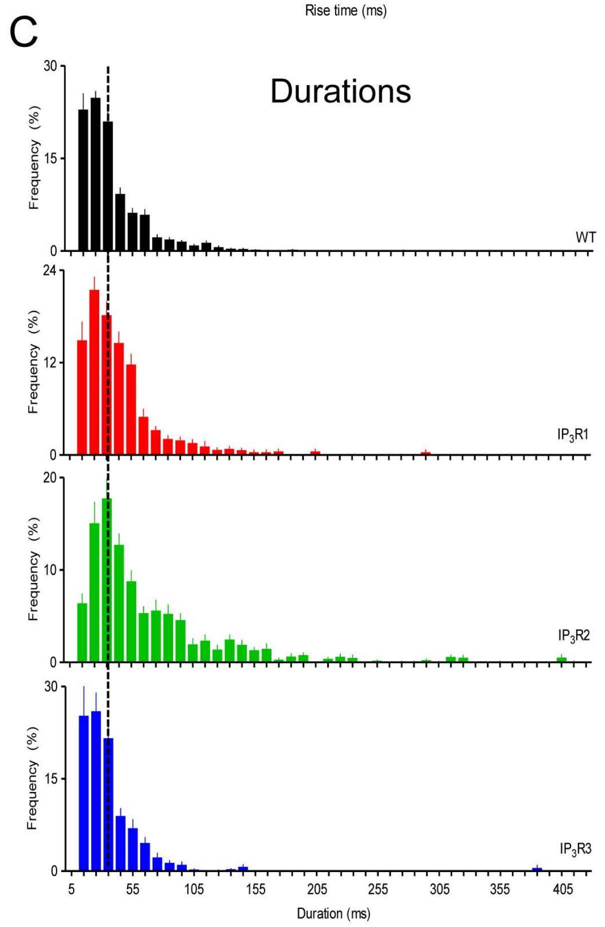

Fig. S4. Frequency distributions of rise times, decay times and durations of Ca^{2+} puffs. (A-C) For Ca^{2+} puffs evoked by photolysis of ci-IP_3 in each cell line, the rise times (A, time for fluorescence to increase from 20% to 100% of peak amplitude), decay times (B, time for fluorescence to fall from 100% to 20% of peak amplitude) and durations at half-maximal amplitude (C) were measured. Mean values for WT cells are shown by dashed lines. Results (mean ± s.e.m., *n* = 15 cells) show the frequency for each 20-ms (A,B) or 10-ms (C) time-bin. Summary data in **Fig. 3A-C**.

Journal of Cell Science • Supplementary information

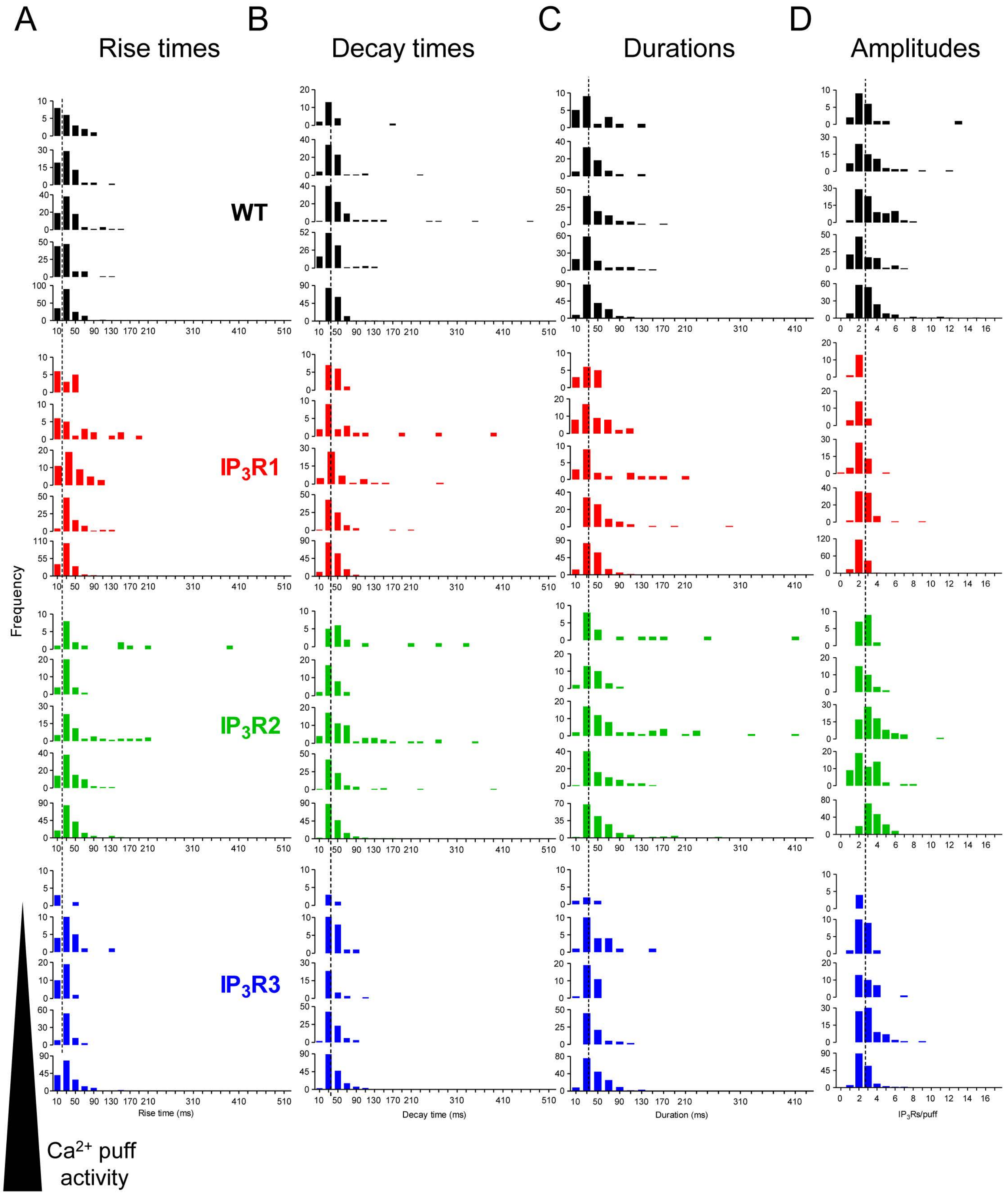

Fig. S5. Distributions of Ca^{2+} puff rise times, decay times, durations and amplitudes in the most and least active cells. (A-D) For each cell line, five cells were selected to represent the range of Ca^{2+} puff activities from the least (top panels) to most active cells (bottom panels). Frequency distributions (20-ms bins) of rise times (A, time for fluorescence to increase from 20% to 100% of maximal value), decay times (B, time for fluorescence to fall from 100% to 20% of maximal value) and durations at half-maximal amplitude (C), and of the estimated number of IP_3Rs in each Ca^{2+} puff (D) are shown for 5 cells selected from each of the 4 cell lines. Dashed lines show the mean values for WT cells.

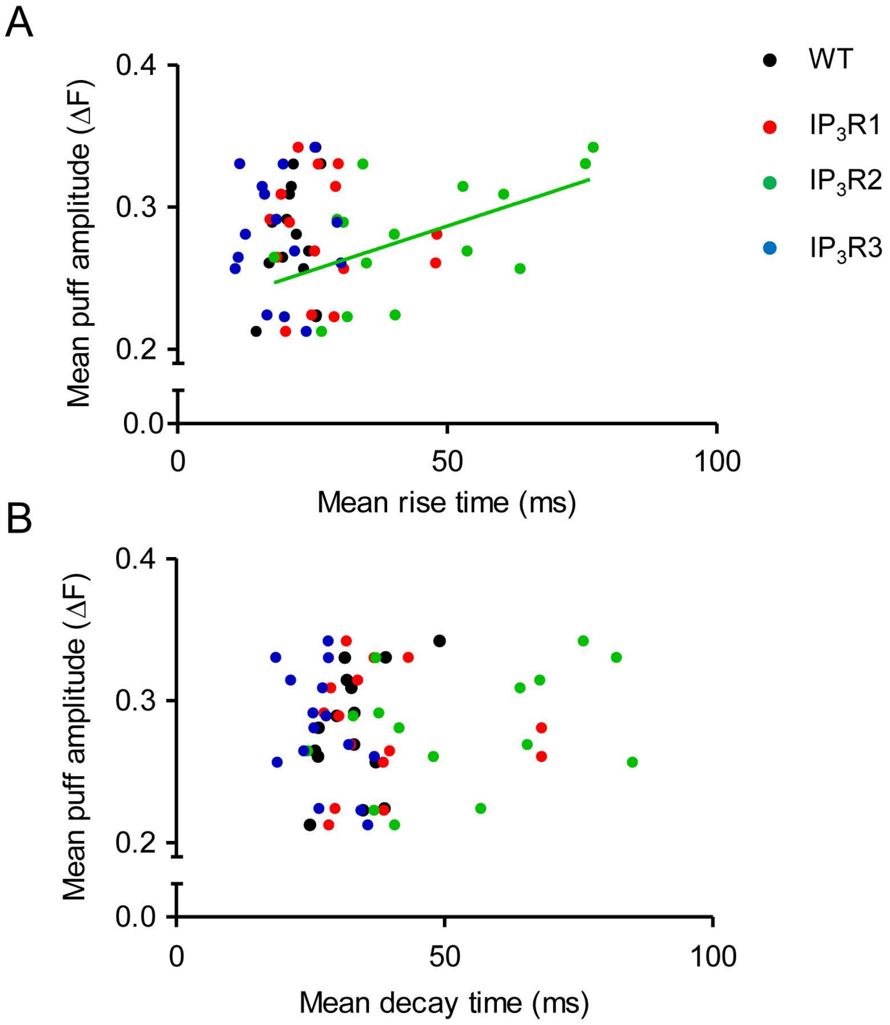

Fig. S6. Relationships between Ca^{2+} puff amplitudes and kinetics. (A,B) For each cell (n = 15) in each of the four cell lines, the relationship between the mean amplitude of the Ca^{2+} puffs (ΔF) and the mean rise (A) and decay (B) times are shown. The only relationship for which the slope differed significantly from zero ($P < 0.05$) is shown.
